# Perceptions, Barriers, and Breakfast Consumption Habits Among University Students in the United Arab Emirates: A Cross-Sectional Study

**DOI:** 10.7759/cureus.115339

**Published:** 2026-08-28

**Authors:** Doaa Q Aljanabi, Omar M Al Ani, Mohamed J Almulla, Sara Rashid, Tayaba Sahibdad, Ahmad R Yacoub, Maryam A Alsane, Mitha Althakhri, Rania S Ahmed, Amal Hussein

**Affiliations:** 1 College of Medicine, University of Sharjah, Sharjah, ARE; 2 Department of Basic Sciences, University of Sharjah, Sharjah, ARE; 3 Department of Family and Community Medicine and Behavioral Sciences, University of Sharjah, Sharjah, ARE

**Keywords:** breakfast barriers, breakfast consumption, breakfast skipping, dietary habits, medical and non-medical students, nutritional knowledge, united arab emirates (uae), university students

## Abstract

Background

University students often skip breakfast due to academic demands and lifestyle habits. However, evidence on breakfast consumption among university students in the United Arab Emirates remains limited, highlighting the need for locally relevant data.

Aim

This study aimed to investigate breakfast consumption patterns among medical and non-medical university students in the UAE, with a particular focus on eating habits, consumption frequency, dietary choices, perceived barriers, energy levels, and the relationship between breakfast consumption and body mass index (BMI).

Methods

A cross-sectional study was conducted among 405 medical and non-medical students aged 18 years and above from universities across the UAE during 2024-2025. Participants were recruited using convenience sampling through social media platforms and in-person invitations, which may affect the generalizability of the findings. Data were collected via a structured questionnaire after obtaining approval from the University of Sharjah Research Ethics Committee. Statistical analyses were performed using SPSS version 29 (IBM Corp., Armonk, NY) and included descriptive statistics, Pearson’s chi-square tests, Mann-Whitney U tests, Spearman’s rank correlation, and multivariable binary logistic regression.

Results

Medical students were significantly more likely to skip breakfast than non-medical students (38.2% vs. 25.2%, p = 0.005), mainly due to limited time and busy morning schedules. Both groups showed similar patterns in breakfast consumption frequency. Moreover, students who ate breakfast frequently (six to seven days per week) reported higher energy levels compared to those who ate breakfast less often. In our sample, BMI showed no significant relationship with breakfast consumption. While students from different majors demonstrated similar levels of knowledge, those who regularly ate breakfast exhibited better knowledge and more positive attitudes toward it. Finally, the findings indicated that greater knowledge about breakfast was associated with a more positive attitude.

Conclusions

The findings showed that breakfast skipping was more common among medical students than non-medical students and that more frequent breakfast consumption was associated with higher self-reported energy levels. Breakfast consumption was not significantly associated with BMI in this sample. Regular breakfast consumption was also associated with higher knowledge and more positive attitudes toward breakfast. These findings highlight the importance of addressing barriers to breakfast consumption among university students, particularly limited time and busy morning schedules. University-based strategies addressing these practical barriers may be considered, although their effectiveness should be evaluated in future intervention studies.

## Introduction

Breakfast is widely recognized as an important component of a healthy diet and has long been associated with physical and cognitive health outcomes. Regular breakfast consumption has been linked to healthier dietary patterns, improved nutritional status, and better overall well-being among university students and young adults [1]. Comparisons between medical and non-medical university students have also shown that although students may possess adequate nutritional knowledge, this knowledge does not always translate into healthy dietary practices [2]. Furthermore, evidence suggests that regular breakfast consumption may be associated with attention, memory, cognitive performance, and academic achievement, highlighting its potential role in supporting learning and daily functioning [3].

Despite these potential benefits, breakfast skipping remains common among university students worldwide. Studies among medical students have consistently reported high rates of breakfast skipping, largely due to demanding academic schedules and limited time in the morning [4]. Similar findings have been reported among undergraduate health sciences students, where breakfast habits were influenced by lifestyle factors, daily routines, and sociodemographic characteristics [5]. More recent research among Jordanian university students has likewise identified breakfast skipping as a frequent behavior, with lack of time and irregular morning schedules being the most commonly reported barriers [6]. Comparable observations have also been made among tertiary students in Ghana, where practical barriers, such as busy schedules and competing priorities, contributed to inconsistent breakfast consumption [7]. Likewise, studies conducted among Saudi university students have demonstrated that attitudes toward breakfast and time-related constraints play important roles in determining breakfast-eating behaviors [8].

In addition to these practical barriers, breakfast consumption has been associated with broader indicators of health and wellbeing. Individuals who regularly consume breakfast generally report better mood, improved wellbeing, and healthier lifestyle behaviors than those who frequently skip it [9]. At the same time, rapid socioeconomic development and changing food environments have contributed to dietary transitions among young adults, particularly in developing and rapidly developing countries, where irregular meal patterns and increased reliance on convenience foods have become increasingly common [10]. However, the relationship between breakfast consumption and health outcomes is complex, as body weight and nutritional status are influenced by multiple behavioral and environmental factors rather than breakfast consumption alone [11].

Medical students may be particularly vulnerable to irregular eating habits because of the demanding nature of their academic programs. While they often demonstrate greater awareness of healthy nutrition, previous studies comparing medical and non-medical students have reported inconsistent findings regarding actual breakfast consumption and its relationship with body mass index (BMI), suggesting that nutritional knowledge alone may not be sufficient to promote healthy eating behaviors [12]. Other research has similarly shown that although university students generally recognize the health benefits of breakfast, many continue to skip it because of competing academic responsibilities, personal routines, and lifestyle factors [13]. Recent evidence has further confirmed that breakfast skipping remains highly prevalent among university students, reinforcing the need to better understand the practical barriers limiting regular breakfast consumption [14]. Moreover, some studies have reported an association between regular breakfast intake and better academic performance, although these findings do not establish a causal relationship [15].

In the United Arab Emirates (UAE), available evidence suggests that breakfast skipping is also relevant among university students. A study examining dietary habits and psychosocial factors among university students in the UAE found that less than half of the participants reported consuming breakfast daily; however, the study focused on broader dietary and psychosocial factors rather than specifically comparing breakfast consumption between medical and non-medical students [16]. Previous UAE research has also examined the relationship between breakfast consumption and academic performance among female high-school students in Abu Dhabi, but evidence specifically addressing breakfast patterns among university students remains limited [17]. Therefore, there is a need for locally relevant research examining breakfast consumption among university students, particularly across different academic disciplines. Comparing medical and non-medical students is relevant because differences in nutritional knowledge, academic demands, and daily schedules may influence breakfast-related behaviors, and previous research has shown that nutritional knowledge does not necessarily translate into healthier dietary practices [2,4,12].

Therefore, the primary objective of this study was to investigate breakfast consumption patterns among medical and non-medical university students in the UAE. The secondary objectives were to examine differences in eating habits, breakfast consumption frequency, dietary choices, perceived barriers, energy levels, nutritional knowledge, attitudes toward breakfast, and the relationship between breakfast consumption and BMI. The findings are expected to contribute to the development of targeted nutrition education and health promotion strategies that encourage healthier dietary behaviors within university settings.

## Materials and methods

Study design and participants

**Figure 1:**
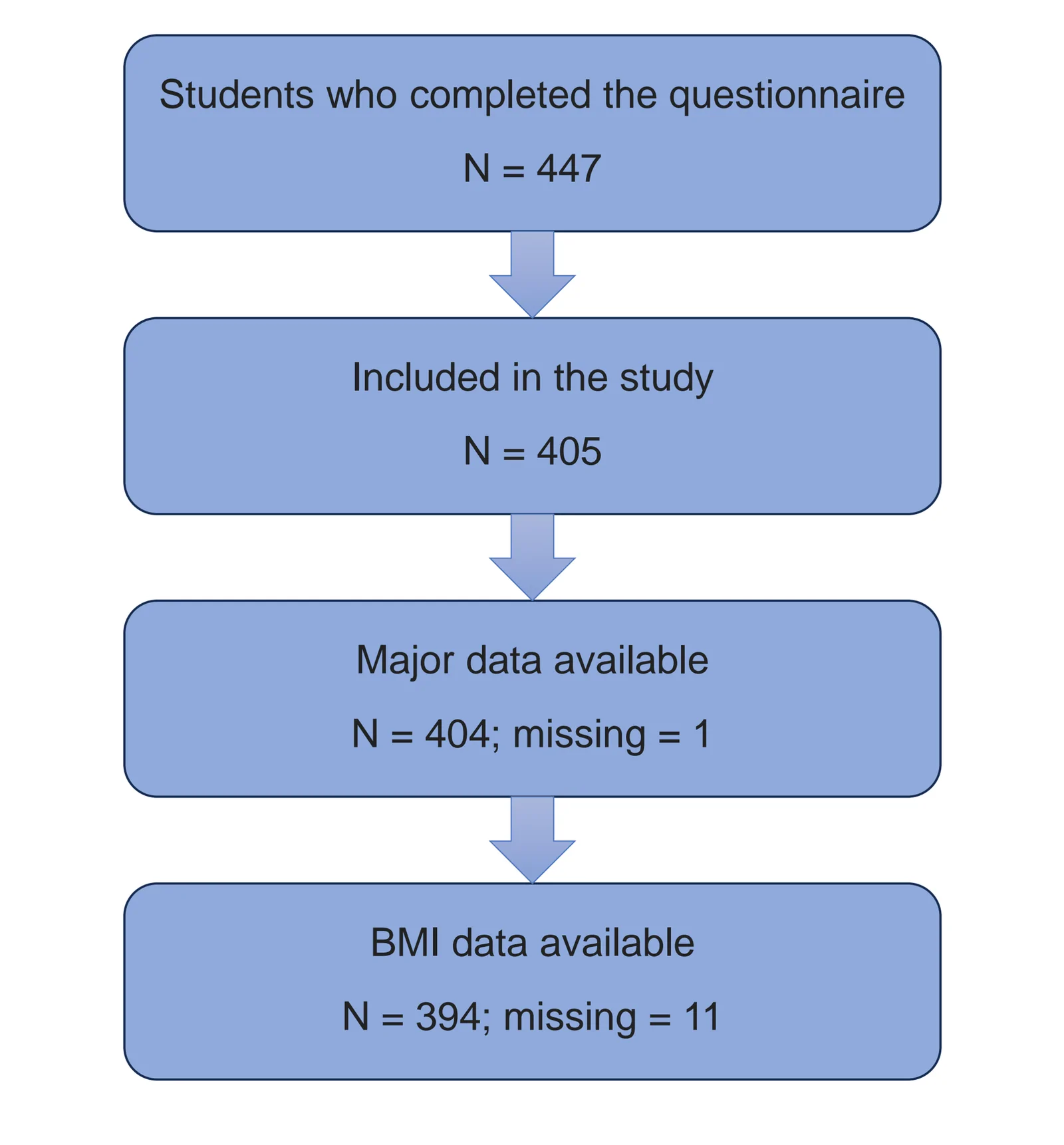
STROBE Flow Diagram of Participant Inclusion in the Study

A descriptive cross-sectional study was conducted in the UAE between February 2024 and November 2025, with data collection conducted between February and March 2025. The study assessed breakfast consumption patterns, eating habits, perceptions, and barriers to breakfast intake among medical and non-medical university students. The participant inclusion process is summarized in Figure 1.

Students aged 18 years and above who were currently enrolled at universities in the UAE were eligible to participate. Both local and expatriate students from medical and non-medical colleges were included. Participants were recruited using convenience sampling through social media platforms and direct in-person invitations at universities across the UAE. Recruitment efforts were distributed across different emirates by members of the research team to obtain broader geographic coverage of university students within the UAE. However, university affiliation and participants’ emirate were not recorded in the questionnaire; therefore, the exact number of participating universities and the distribution of participants by emirate could not be determined. Approximately 550 students were approached, and 447 questionnaire responses were received, corresponding to an approximate response rate of 81.3%.

Students were excluded if they did not provide consent, were under 18 years of age, were not currently enrolled at a university in the UAE, had a diagnosed eating disorder, or were on medical leave from university. After applying the eligibility criteria, 42 responses were excluded, leaving 405 participants in the final analysis.

Because convenience sampling was used, the study sample may not be fully representative of all university students in the UAE, and the findings should be interpreted with consideration of potential selection bias and limited generalizability.

Sample size

The required sample size was determined using Cochran's formula for cross-sectional studies, $n = \frac{Z^{2}p(1 - p)}{d^{2}}$, with a 95% confidence level (Z = 1.96), a margin of error of 5% (d = 0.05), and an expected prevalence of 50% (p = 0.50). The prevalence of 50% was selected as a conservative estimate in the absence of a reliable prior prevalence estimate for the target population, as it provides the largest required sample size. Based on these assumptions, the minimum required sample size was 385 participants. A finite population correction was not applied because the study targeted university students across the UAE rather than a defined finite institutional population. A total of 405 eligible participants were included in the final analysis.

Questionnaire design and data collection

A structured self-administered questionnaire was used for data collection (Appendices). The questionnaire was adapted from a previously published study examining breakfast consumption patterns, knowledge, attitudes, practices, and barriers among university students [1], with modifications made to suit the UAE university context and the objectives of the present study. In the original study, the knowledge, attitudes, and practices questionnaire was pre-tested among 30 university students, and internal consistency was assessed using Cronbach’s alpha. The reported coefficients were 0.722 for knowledge, 0.705 for attitude, and 0.784 for practice, indicating acceptable internal consistency [1]. The original study reported pre-testing and reliability assessment but did not report a separate construct-validity analysis.

For the present study, content validity was assessed through review by the two study supervisors, who examined the questionnaire for clarity, relevance, and appropriateness to the study objectives and UAE university context. The final version was piloted among 10 university students before data collection to assess clarity and feasibility. Feedback from the pilot was used to refine the wording and overall structure of the questionnaire, and pilot responses were excluded from the final analysis. A separate construct-validity analysis was not performed. The internal consistency of the breakfast knowledge items was evaluated using Cronbach’s alpha, yielding a coefficient of 0.74, indicating acceptable internal consistency.

The questionnaire included 12 perception and knowledge statements (Q17-Q28), with response options of “True,” “False,” and “Not sure.” For analysis, each item was scored according to the predetermined correct response. A correct response was assigned a score of 1, whereas an incorrect or “Not sure” response was assigned a score of 0. The item scores were summed to produce an overall knowledge score ranging from 0 to 12, with higher scores indicating greater knowledge.

The attitude score was derived from two items in the health and well-being section assessing participants’ perceptions of the effects of breakfast on alertness and energy levels. The questionnaire used a five-point Likert scale ranging from “strongly disagree” to “strongly agree” for these two items. For scoring, “agree” and “strongly agree” responses were assigned a score of 1, whereas “neutral,” “disagree,” and “strongly disagree” responses were assigned a score of 0. The two item scores were summed to produce an overall attitude score ranging from 0 to 2, with higher scores indicating more positive perceptions of the effects of breakfast on alertness and energy levels.

The final questionnaire comprised six sections: consent, sociodemographic characteristics, breakfast consumption patterns, perceptions and knowledge related to breakfast, barriers to breakfast consumption, and the perceived impact of breakfast on health and well-being. The sociodemographic section included gender, age, academic major, height, and weight, from which BMI was calculated and categorized using standard classifications, consistent with prior studies [1]. The breakfast consumption section assessed consumption status, frequency, timing, eating location, eating companions, and usual breakfast food choices. The questionnaire also assessed barriers to breakfast consumption and participants’ perceptions of the effects of breakfast on alertness and energy levels.

Statistical analysis

Data were analyzed using SPSS version 29 (IBM Corp., Armonk, NY). Before analysis, the dataset was reviewed for completeness, consistency, and potential data-entry errors.

Descriptive statistics were used to summarize participant characteristics and the main study variables. Categorical variables, including gender, age group, academic major, breakfast consumption status, breakfast consumption frequency, breakfast timing, breakfast location, breakfast-related barriers, and BMI categories, were summarized using frequencies and percentages. Continuous variables, including knowledge and attitude scores, were presented as means and standard deviations.

The distribution of the knowledge and attitude scores was assessed for normality using the Shapiro-Wilk test and visual inspection of Q-Q plots. Both scores showed statistically significant departures from normality (p < 0.001); therefore, non-parametric tests were used for analyses involving these scores. The Shapiro-Wilk statistics were 0.906 for the knowledge score and 0.801 for the attitude score, with p < 0.001 for both (N = 405).

Inferential analyses were performed to examine associations between study variables. Pearson's chi-square test was used to assess relationships between categorical variables, including academic major and breakfast consumption status, breakfast consumption frequency, breakfast timing, BMI categories, and self-reported energy levels. The chi-square assumption regarding expected cell frequencies was assessed, and expected counts were reviewed for the categorical analyses. Cramér's V was used to report the effect size for chi-square associations. The Mann-Whitney U test was used to compare knowledge and attitude scores between medical and non-medical students and between breakfast consumers and breakfast skippers. Spearman's rank correlation was used to evaluate the relationship between knowledge and attitude scores. The Mann-Whitney analyses included 404 participants for comparisons by academic major and 405 participants for comparisons by breakfast consumption status. The Spearman correlation analysis included 405 participants.

A multivariable binary logistic regression analysis was performed to identify factors independently associated with breakfast skipping. Academic major, BMI category, knowledge score, and attitude score were entered simultaneously into the model. Adjusted odds ratios (AORs) with 95% confidence intervals (CIs) were reported. Model fit was assessed using the Hosmer-Lemeshow goodness-of-fit test. The regression analysis included 393 participants with complete data for all variables included in the model.

Cases with missing data were excluded only from the relevant analyses, while all other available data were retained. Specifically, BMI analyses were conducted using data from 394 participants because 11 participants did not provide complete height and/or weight information required for BMI calculation. Academic-major analyses were based on 404 participants because academic-major information was missing for one participant.

Given that the inferential analyses addressed distinct prespecified associations rather than repeated pairwise comparisons within a single hypothesis family, no formal adjustment for multiple comparisons was applied. All statistical tests were two-tailed, and a p-value of less than 0.05 was considered statistically significant.

Ethical considerations

Ethical approval for this study was obtained from the Research Ethics Committee of the University of Sharjah prior to data collection (REC-25-03-16-41-S). Participation was voluntary, and electronic informed consent was obtained from all participants before completion of the questionnaire. Participant confidentiality was maintained throughout the study.

## Results

Participant characteristics

A total of 405 university students from different universities across the UAE participated in this study. Academic-major data were available for 404 participants, of whom 186 (46.0%) were medical students and 218 (54.0%) were non-medical students; one participant had missing major data. Among participants with available academic-major data, 141 (34.9%) were male, and 263 (65.1%) were female. Most participants were aged 18-20 years (n = 246, 60.9%), followed by 21-23 years (n = 127, 31.4%), 24-26 years (n = 10, 2.5%), and ≥27 years (n = 21, 5.2%). Among the 393 participants with complete data for both academic major and BMI category, 224 (57.0%) had normal weight, 93 (23.7%) were overweight, 39 (9.9%) were underweight, and 37 (9.4%) were obese. When participant characteristics were compared by academic major, gender and age distributions differed significantly between medical and non-medical students (both p < 0.001), whereas BMI category did not differ significantly between the two groups (p = 0.394). The detailed characteristics of the participants according to academic major are summarized in Table 1. 

**Table 1:** General Characteristics of the Participants (N = 405) *Significant p-values (p < 0.05) were calculated using SPSS. Values are presented as n (%). Percentages within the medical and non-medical groups are column percentages. p-values were calculated using Pearson’s chi-square test. Academic-major data were available for 404 participants; one participant had missing major data. The BMI comparison by academic major included 393 participants with complete data for both variables. BMI was calculated based on self-reported height and weight. Complete BMI data were available for 394 participants, as 11 respondents did not provide complete height and/or weight information. These cases were excluded only from BMI-related analyses, and BMI percentages for the overall sample were calculated using the 394 participants with complete BMI data.

Variable	Category	Medical n (%)	Non-medical n (%)	Total n (%)	p-value
Gender	Male	46 (24.7)	95 (43.6)	141 (34.9)	<0.001*
	Female	140 (75.3)	123 (56.4)	263 (65.1)	-
Age	18-20 years	115 (61.8)	131 (60.1)	246 (60.9)	<0.001*
	21-23 years	70 (37.6)	57 (26.1)	127 (31.4)	-
	24-26 years	1 (0.5)	9 (4.1)	10 (2.5)	-
	≥27 years	0 (0.0)	21 (9.6)	21 (5.2)	-
BMI	Underweight	16 (8.8)	23 (10.8)	39 (9.9)	0.394
	Normal weight	105 (58.0)	119 (56.1)	224 (57.0)	-
	Overweight	47 (26.0)	46 (21.7)	93 (23.7)	-
	Obese	13 (7.2)	24 (11.3)	37 (9.4)	-

Breakfast consumption patterns

Overall, 279 (68.9%) of the 405 participants reported consuming breakfast regularly, while 126 (31.1%) reported skipping breakfast. Among the 404 participants with available academic-major data, 278 (68.8%) were regular breakfast consumers and 126 (31.2%) skipped breakfast. Breakfast consumption differed significantly by academic major (χ²(1) = 7.834, p = 0.005). Medical students reported a higher prevalence of breakfast skipping (71, 38.2%) compared with non-medical students (55, 25.2%). The odds of skipping breakfast were 1.83 times higher among medical students than among non-medical students (OR = 1.83, 95% CI: 1.20-2.80). In contrast, 163 (74.8%) non-medical students reported regularly consuming breakfast compared with 115 (61.8%) medical students. A comparison of breakfast consumption between medical and non-medical students is presented in Table 2.

**Table 2:** Comparison of Breakfast Consumption by Academic Major (N = 404) *Significant p-values (p < 0.05) were calculated using SPSS. Values are presented as n (%), with percentages calculated within academic-major groups. The p-value was calculated using Pearson’s chi-square test. OR, odds ratio; CI, confidence interval. The OR represents the odds of breakfast skipping among medical students compared with non-medical students. Academic-major data were available for 404 participants; one participant had missing academic-major data. Statistically significant at p < 0.05.

Variable	Medical Students, n (%)	Non-medical Students, n (%)	Total, n (%)	OR (95% CI)	p-value
Regular breakfast consumers	115 (61.8)	163 (74.8)	278 (68.8)	-	-
Skip breakfast	71 (38.2)	55 (25.2)	126 (31.2)	1.83 (1.20-2.80)	0.005*
Total	186 (100.0)	218 (100.0)	404 (100.0)	-	-

Although medical students skipped breakfast more often than non-medical students, the difference in breakfast consumption frequency between the two groups was not statistically significant (p = 0.459). In both groups, the most commonly reported breakfast consumption frequency was two to three times per week (135, 33.3%), followed by six to seven times per week (110, 27.2%). Smaller proportions of participants reported eating breakfast four to five times per week (81, 20.0%) or zero or one time per week (79, 19.5%). Similarly, the majority of both medical and non-medical students reported consuming breakfast between 8:00 AM and 11:00 AM (202, 49.9%), with no statistically significant difference in breakfast timing between the two groups (p = 0.287).

Most participants reported consuming breakfast at home (343, 84.7%), while fewer ate at cafeterias (149, 36.8%) or restaurants (96, 23.7%). Regarding breakfast items, eggs were the most commonly consumed food (271, 66.9%), followed by tea or coffee (237, 58.5%), sandwiches (219, 54.1%), and chocolate (196, 48.4%). Other commonly reported items included bread or toast (180, 44.4%) and cereal or oatmeal (142, 35.1%). In contrast, healthier options such as fruits (111, 27.4%) and yogurt (87, 21.5%) were less frequently consumed among both medical and non-medical students. Participants were allowed to select more than one breakfast location and more than one breakfast item; therefore, these percentages are not mutually exclusive and may total more than 100%. Table 3 summarizes the reported breakfast locations and the commonly consumed breakfast items among the study participants.

**Table 3:** Breakfast Location and Commonly Consumed Breakfast Items Among Participants (N = 405) Multiple responses were permitted for breakfast location and breakfast items; therefore, percentages may exceed 100%. Percentages were calculated using the total sample (N = 405).

Breakfast Characteristics	n (%)
Breakfast location	
Home	343 (84.7)
Cafeteria	149 (36.8)
Restaurant	96 (23.7)
Breakfast items consumed	
Eggs	271 (66.9)
Tea/coffee	237 (58.5)
Sandwiches	219 (54.1)
Chocolate	196 (48.4)
Bread/toast	180 (44.4)
Cereal/oatmeal	142 (35.1)
Fruits	111 (27.4)
Yogurt	87 (21.5)

Breakfast perceptions and knowledge

Overall, participants showed good knowledge of several aspects of breakfast. Most recognized that breakfast provides vitamins and minerals (379, 93.6%), improves mood and academic performance (344, 84.9%), enhances concentration and helps retain information in class (331, 81.7%), and supplies glucose to the brain after an overnight fast (322, 79.5%). Many participants also identified the recommended breakfast time as between 6:00 AM and 10:00 AM (314, 77.5%) and agreed that a high-fiber breakfast may reduce the risk of heart disease (272, 67.2%). More than half believed that regular breakfast consumption may help reduce body weight (231, 57.0%). For the statement that breakfast increases calorie intake at the next meal, 111 (27.4%) participants responded “True,” 170 (42.0%) responded “False,” and 123 (30.4%) were unsure. Just over half of the participants disagreed with the statement that a breakfast high in fiber and protein but low in fat and sugar reduces concentration during learning (223, 55.1%). Table 4 presents the participants’ perceptions and knowledge regarding breakfast.

**Table 4:** Participants’ Perceptions/Knowledge Regarding Breakfast (N = 405) Percentages are reported to one decimal place for consistency. Q17, Q20, and Q28 each had one missing original response; therefore, the response-category percentages for these items may not sum to 100.0%. Minor differences from 100.0% may also occur because of rounding.

Perceptions/Knowledge Questions	True, n (%)	False, n (%)	Not Sure, n (%)
Breakfast increases calorie intake at next meal	111 (27.4)	170 (42.0)	123 (30.4)
Breakfast provides vitamins and minerals	379 (93.6)	8 (2.0)	18 (4.4)
High-fiber breakfast increases satiety	331 (81.7)	22 (5.4)	52 (12.8)
Breakfast improves concentration and memory	331 (81.7)	26 (6.4)	47 (11.6)
Breakfast leads to unhealthy snacking	60 (14.8)	272 (67.2)	73 (18.0)
A breakfast high in fiber and protein but low in fat and sugar reduces concentration	90 (22.2)	223 (55.1)	92 (22.7)
Regular breakfast helps reduce body weight	231 (57.0)	50 (12.3)	124 (30.6)
High-fiber breakfast reduces heart disease risk	272 (67.2)	21 (5.2)	112 (27.7)
The best time to eat breakfast is between 6:00 AM and 10:00 AM	314 (77.5)	23 (5.7)	68 (16.8)
Breakfast provides glucose to the brain after an overnight fast	322 (79.5)	15 (3.7)	68 (16.8)
Eating breakfast improves mood and academic performance	344 (84.9)	14 (3.5)	47 (11.6)
A high-fiber breakfast helps improve digestion	305 (75.3)	21 (5.2)	78 (19.3)

The 12 knowledge items (Q17-Q28) were dichotomously scored as 0 or 1 according to the correct response. Correct responses were assigned a score of 1, while incorrect and “Not sure” responses were assigned a score of 0. The item scores were summed to produce a total knowledge score with a possible range of 0 to 12, with higher scores indicating greater knowledge. The observed knowledge scores also ranged from 0 to 12. Three knowledge items (Q17, Q20, and Q28) each had one missing original response. For calculation of the total knowledge score, these missing responses were coded as 0 in the corresponding dichotomous item scores. No additional imputation was performed.

There was no statistically significant difference in knowledge scores between medical and non-medical students (p > 0.05). Although medical students had a slightly higher mean knowledge score (M = 8.15) compared with non-medical students (M = 7.74), the difference was not statistically significant.

Barriers to breakfast consumption

The main barriers to breakfast consumption were similar in both groups. Limited time was the most commonly reported reason for skipping breakfast, 240 (59.3%), followed by waking up late, 128 (31.6%), mood-related factors, 124 (30.6%), and feeling that it was too early to eat, 115 (28.4%). Participants were able to report more than one barrier; therefore, percentages are not mutually exclusive and may exceed 100%. Table 5 presents the barriers related to breakfast consumption among medical and non-medical students.

**Table 5:** Barriers Related to Breakfast Consumption (N = 405) Multiple responses were permitted for barriers to breakfast consumption; therefore, percentages may exceed 100%. Percentages were calculated using the total sample (N = 405).

Variable	n (%)
Due to my mood	124 (30.6)
Because of a health condition	5 (1.2)
Weight management	51 (12.6)
Limited time	240 (59.3)
Daily activities or workload	75 (18.5)
Cost of food on campus	37 (9.1)
Too early to eat	115 (28.4)
Wake up too late	128 (31.6)
Breakfast is not important	41 (10.1)

Associations between variables

A significant association was observed between students’ field of study and breakfast consumption (χ²(1) = 7.834, p = 0.005; N = 404), indicating that breakfast consumption differed between medical and non-medical students. Medical students had higher odds of skipping breakfast than non-medical students (unadjusted OR = 1.83, 95% CI: 1.20-2.80). Breakfast consumption was also significantly associated with self-reported energy levels (χ²(4) = 52.943, p < 0.001, Cramér’s V = 0.362; N = 405), with students who regularly consumed breakfast tending to report higher energy levels than those who skipped breakfast. Similarly, breakfast frequency was significantly associated with energy levels (χ²(12) = 83.961, p < 0.001, Cramér’s V = 0.263; N = 405), with more frequent breakfast consumption associated with higher self-reported energy levels.

No significant association was found between breakfast consumption and BMI category (χ²(3) = 3.855, p = 0.278, Cramér’s V = 0.099; N = 394). This analysis was based on the 394 participants with available BMI data.

Knowledge scores differed significantly according to breakfast consumption status (Mann-Whitney U = 14,083.000, Z = -3.237, p = 0.001, r = 0.161; N = 405), with regular breakfast consumers having a higher mean rank than those who skipped breakfast (215.52 vs. 175.27). Attitude scores also differed significantly between the two groups (Mann-Whitney U = 11,108.000, Z = -6.324, p < 0.001, r = 0.314; N = 405), with regular breakfast consumers having a higher mean rank than those who skipped breakfast (226.19 vs. 151.66).

A significant positive correlation was observed between knowledge and attitude scores (Spearman’s ρ = 0.346, p < 0.001; N = 405), indicating that students with greater knowledge about breakfast tended to have more favorable attitudes toward breakfast consumption. Table 6 summarizes the associations between the study variables.

**Table 6:** Summary of Statistical Associations *Significant p-values (p < 0.05) were calculated using SPSS. Statistically significant at p < 0.05. Cramér’s V and r are reported as effect-size measures. Analysis-specific sample sizes (N) are reported because data availability differed across variables.

Variable Relationship	Statistical Test	Test Value	p-value	Effect Size/Estimate (95% CI, Where Applicable)	N
Breakfast consumer × Major	Chi-square test	χ²(1) = 7.834	0.005*	OR = 1.83 (95% CI: 1.20-2.80)	404
Breakfast consumer × BMI category	Chi-square test	χ²(3) = 3.855	0.278	Cramér’s V = 0.099	394
Breakfast consumer × Energy	Chi-square test	χ²(4) = 52.943	<0.001*	Cramér’s V = 0.362	405
Breakfast frequency × Energy	Chi-square test	χ²(12) = 83.961	<0.001*	Cramér’s V = 0.263	405
Breakfast consumer × Knowledge score	Mann-Whitney U test	U = 14,083.000; Z = -3.237	0.001*	r = 0.161	405
Breakfast consumer × Attitude score	Mann-Whitney U test	U = 11,108.000; Z = -6.324	<0.001*	r = 0.314	405
Knowledge score × Attitude score	Spearman correlation	ρ = 0.346	<0.001*	ρ = 0.346	405

Final results summary

Overall, medical students had higher odds of skipping breakfast than non-medical students. Regular breakfast consumption and greater breakfast frequency were significantly associated with higher self-reported energy levels, whereas breakfast consumption was not significantly associated with BMI category. Regular breakfast consumers also had significantly higher knowledge and attitude ranks than students who skipped breakfast. In addition, greater breakfast knowledge was positively associated with more favorable attitudes toward breakfast.

## Discussion

The present study explored breakfast consumption patterns, perceptions, and barriers among university students in the UAE, comparing medical and non-medical students. Overall, breakfast skipping emerged as a frequent behavior, with medical students showing a noticeably higher tendency to skip breakfast than their non-medical counterparts. This difference may reflect differences in academic and daily routines, although the specific reasons for this difference were not directly examined in this study. Similar patterns have been documented in various settings, where academic pressure, time constraints, and irregular sleep schedules are consistently reported as key reasons for missing breakfast among students [4-8,14]. Broader evidence from studies on university populations also supports the idea that lifestyle structure plays a major role in dietary behavior, particularly during late adolescence and early adulthood [1,2,16].

Although differences were observed in actual breakfast consumption, students from both groups demonstrated generally good knowledge regarding the importance of breakfast. Most participants recognized that eating breakfast can enhance concentration, mood, and energy levels throughout the day. However, this study highlights a clear gap between knowledge and practice, as awareness did not consistently translate into regular breakfast consumption. This disconnect has been widely reported in the literature, where students often understand the benefits of healthy eating but struggle to apply them in daily life due to competing academic and personal priorities [1,2,9,13,16]. Similar findings have also been observed in broader nutritional studies, which suggest that even in populations with adequate nutritional awareness, behavioral change is often limited by environmental and lifestyle constraints [1,7]. In addition, wider dietary shifts among young people, particularly in changing food environments, may further contribute to inconsistent eating patterns [10].

A key finding of this research was the association between regular breakfast consumption and higher self-reported energy levels. Students who ate breakfast more consistently reported feeling more alert and energetic throughout the day compared to those who frequently skipped it. This aligns with existing evidence suggesting that breakfast intake is associated with cognitive function, attention, and overall academic performance in young adults [3,15,17]. Other studies have similarly highlighted that breakfast consumption is linked with better perceived wellbeing and daily functioning, reinforcing its role as an important component of students’ routines [9,13]. Recent evidence has also shown that students who regularly skip breakfast may experience poorer academic performance, further emphasizing the importance of maintaining consistent breakfast habits during education [15,17]. However, given the cross-sectional design of the present study, this association should not be interpreted as evidence that breakfast consumption directly increases energy levels. Other factors, such as sleep, stress, and academic workload, may also influence perceived energy levels.

Interestingly, no significant relationship was found between breakfast consumption and BMI among the 394 participants with available BMI data. BMI data were unavailable for 11 participants because of incomplete self-reported height and/or weight information; therefore, these participants were excluded from BMI-related analyses. This suggests that body weight may not be strongly related to breakfast habits alone, but rather to a combination of multiple lifestyle factors such as overall diet quality, physical activity, sleep patterns, and stress levels. Previous research has also reported inconsistent or weak associations between breakfast intake and BMI in university populations [12]. A systematic review and meta-analysis of randomized controlled trials also found no clear effect of breakfast consumption on body weight, suggesting that the relationship between breakfast consumption and weight-related outcomes remains uncertain [18]. Differences between studies may be related to variations in study populations, definitions of breakfast consumption or skipping, dietary patterns, physical activity levels, and methods of BMI assessment. Broader evidence further supports the idea that weight status is shaped by complex behavioral and environmental influences rather than a single eating habit [10,11]. For example, dietary transitions and changing eating patterns among young populations have been shown to affect overall nutrition quality more broadly than isolated meal patterns [10]. In the present study, detailed dietary intake and physical activity were not assessed, which may also have limited the ability to detect an association between breakfast consumption and BMI. The BMI findings should therefore be interpreted in the context of the reduced analysis sample and the use of self-reported height and weight.

Furthermore, an important finding was the positive relationship between knowledge and attitude scores toward breakfast consumption. Students with better nutritional knowledge tended to show more favorable attitudes toward maintaining regular breakfast habits. This finding supports previous research indicating that knowledge is an important foundation for shaping dietary attitudes, even if it does not always guarantee behavioral change [1,2]. Studies among university students in different regions have similarly shown that while awareness of healthy eating is relatively high, actual practice is often influenced by barriers such as time constraints, academic pressure, and lifestyle habits [7,8,14,16]. Research on breakfast beliefs and health perceptions also suggests that attitudes toward breakfast are closely linked to perceived health benefits and personal experiences [9,13]. The coexistence of good knowledge and favorable attitudes with frequent breakfast skipping highlights the importance of addressing the knowledge-practice gap rather than focusing on awareness alone.

Taken together, the findings of this study highlight the need for targeted interventions to improve breakfast consumption among university students in the UAE, particularly within medical programs where breakfast skipping was more frequently reported. Addressing practical challenges such as time management and accessibility of healthy breakfast options may be more effective than focusing on awareness alone. Previous studies have emphasized that improving dietary behaviors among students requires a combination of education, environmental support, and lifestyle-friendly strategies rather than knowledge enhancement in isolation [1,2,7,14]. A peer-support intervention among Japanese college students was associated with improvements in breakfast frequency and composition, suggesting that structured, peer-based approaches may have potential in addressing breakfast-related behaviors [19]. More broadly, healthy dietary patterns are associated with improved health, active lifestyles, and wellbeing [20]. Strengthening such approaches may ultimately contribute to better energy levels, improved wellbeing, and more consistent healthy eating patterns among students.

In addition to these findings, this study provides valuable insight into breakfast consumption among university students in the UAE by examining not only eating habits, but also perceptions, barriers, energy levels, and BMI. The inclusion of both medical and non-medical students allowed for comparisons between different academic backgrounds, while the relatively large sample size strengthened the reliability of the findings. In addition, the questionnaire covered multiple aspects of breakfast consumption, providing a broader understanding of the factors that may influence students’ breakfast behaviors. The inclusion of students from the UAE also adds to the regional evidence on dietary habits and breakfast-related outcomes among students [16,17].

However, several limitations should be acknowledged. The cross-sectional nature of the study limits the ability to determine causal relationships between breakfast consumption and the outcomes assessed. Furthermore, participants were recruited using convenience sampling, which may have introduced selection bias and limited the representativeness of the sample, thereby limiting the external validity of the findings. The study also relied on self-reported information, including dietary habits, height, and weight, which may be affected by recall bias, inaccurate reporting, and social desirability bias. Additionally, detailed dietary intake was not assessed, and factors such as sleep quality, stress levels, physical activity, socioeconomic status, and living arrangements were not explored. These factors could have influenced breakfast consumption and the outcomes assessed, resulting in residual confounding. The lack of physical activity and sleep assessments is particularly relevant when interpreting the findings related to energy levels and BMI. Therefore, the findings should be interpreted with caution when generalizing them to the wider university student population in the UAE, and causal conclusions should be avoided.

Despite these limitations, this study contributes to a better understanding of breakfast habits among university students in the UAE. The findings emphasize the importance of considering students’ daily challenges when promoting healthier eating behaviors and highlight the need for practical approaches that can help students maintain more regular breakfast patterns.

## Conclusions

This study examined breakfast consumption patterns, perceptions, and barriers among medical and non-medical university students in the UAE. Breakfast skipping was more common among medical students, with limited time and demanding academic schedules identified as the most frequently reported barriers. Regular breakfast consumption was associated with higher self-reported energy levels and more positive attitudes toward breakfast. Given the cross-sectional nature of the study, these findings represent associations and do not establish a causal relationship.

These findings highlight the potential value of practical strategies that support healthy eating habits among university students. University-based initiatives, including nutrition education, greater access to healthy breakfast options on campus, and measures that help students overcome common barriers to breakfast consumption, could be considered as approaches to support regular breakfast consumption; however, their effectiveness should be evaluated in future studies. Further longitudinal and interventional research is needed to clarify the direction of the observed associations and to examine the potential influence of factors such as sleep, stress, physical activity, and academic schedules on breakfast habits and related health outcomes.

## Appendices

Questionnaire used in the study 

**Table 7:** Participant Information and Consent

Item	Response
Participant information	Participants were informed that the study investigates breakfast consumption, perceptions, barriers, eating habits, and their association with body mass index (BMI) among medical and non-medical university students in the UAE. Participation was voluntary, anonymous, and confidential, and completion of the questionnaire required approximately 5-10 minutes.
Consent	I agree to participate in this study

**Table 8:** Sociodemographic Characteristics *Participants answering "Yes" to either of these questions were excluded from further participation.

Question	Response Options
Gender	☐ Male ☐ Female
Last three letters of your last name	Open response
Last three digits of your mobile phone number	Open response
Age group	☐ 18-20 years ☐ 21-23 years ☐ 24-26 years ☐ ≥27 years
Body weight (kg)	Open response
Height (cm)	Open response
Major	☐ Medical ☐ Non-medical
If you are a medical student, indicate your stage	☐ Pre-clinical ☐ Clinical
Have you been diagnosed with an eating disorder?*	☐ Yes ☐ No
Are you currently on medical leave from the university?*	☐ Yes ☐ No

**Table 9:** Breakfast Consumption Patterns

Question	Response Options
Are you a breakfast consumer?	☐ Yes ☐ No
How often do you eat breakfast each week?	☐ 0-1 times ☐ 2-3 times ☐ 4-5 times ☐ 6-7 times
What time do you usually eat breakfast?	☐ Before 8:00 am ☐ 8:00-11:00 am ☐ After 11:00 am ☐ I do not usually eat breakfast
Where do you usually eat breakfast? (Multiple responses allowed)	☐ University cafeteria ☐ Restaurant/Food stall ☐ Home/Hostel
With whom do you usually eat breakfast? (Optional)	☐ With friends ☐ Alone
What foods do you usually eat for breakfast? (Multiple responses allowed)	☐ Cereal/Oats ☐ Eggs ☐ Bread/Toast ☐ Fruits ☐ Yoghurt ☐ Sandwiches ☐ Chocolate ☐ Coffee/Tea ☐ Other (specify)

**Table 10:** Perceptions Regarding Breakfast

Statement	Response Options
Breakfast consumption increases calorie intake at the next meal.	☐ True ☐ False ☐ Not sure
Breakfast provides important vitamins and minerals.	☐ True ☐ False ☐ Not sure
High-fiber breakfasts help me feel full for longer.	☐ True ☐ False ☐ Not sure
Eating breakfast helps students concentrate and retain new information during class.	☐ True ☐ False ☐ Not sure
Eating breakfast leads to greater consumption of unhealthy snacks.	☐ True ☐ False ☐ Not sure
A breakfast high in fiber and protein but low in fat and sugar reduces concentration during learning.	☐ True ☐ False ☐ Not sure
Regular consumption of a healthy breakfast helps reduce body weight.	☐ True ☐ False ☐ Not sure
Eating a high-energy, high-fiber breakfast reduces the risk of heart disease.	☐ True ☐ False ☐ Not sure
The best time to eat breakfast is between 6:00 am and 10:00 am.	☐ True ☐ False ☐ Not sure
Breakfast provides glucose that helps the brain function after overnight fasting.	☐ True ☐ False ☐ Not sure
Eating breakfast improves mood and academic performance.	☐ True ☐ False ☐ Not sure
A high-fiber breakfast helps improve digestion.	☐ True ☐ False ☐ Not sure

**Table 11:** Barriers to Breakfast Consumption

Question	Response Options (Multiple Responses Allowed)
What are the main reasons you skip breakfast?	☐ Mood ☐ Health condition ☐ Weight management ☐ Limited time/Unfavorable schedule ☐ Daily activities or workload ☐ Cost of food on campus ☐ Too early to eat ☐ Wake up too late ☐ Breakfast is not important ☐ Other (specify)

**Table 12:** Impact of Breakfast on Health and Well-Being

Statement	Response Scale
I am not fully alert until I have had breakfast.	☐ Strongly disagree ☐ Disagree ☐ Neutral ☐ Agree ☐ Strongly agree
My general energy levels are better on days when I eat breakfast.	☐ Strongly disagree ☐ Disagree ☐ Neutral ☐ Agree ☐ Strongly agree
